# Social support as a mediator in the relationship between technostress or academic stress and health: analysis by gender among university students

**DOI:** 10.3389/fpsyg.2023.1236825

**Published:** 2023-09-07

**Authors:** Angela Asensio-Martínez, Alejandra Aguilar-Latorre, Bárbara Masluk, Santiago Gascón-Santos, María Antonia Sánchez-Calavera, Raquel Sánchez-Recio

**Affiliations:** ^1^Department of Psychology and Sociology, University of Zaragoza, Zaragoza, Spain; ^2^Aragonese Primary Care Research Group (GAIAP), Institute for Health Research Aragón (IIS Aragón), Zaragoza, Spain; ^3^Research Network on Chronicity, Primary Care and Health Promotion (RICAPPS, RD21/0016/0005), Carlos III Health Institute, Madrid, Spain; ^4^Department of Medicine, Psychiatry and Dermatology, University of Zaragoza, Zaragoza, Spain; ^5^Health Center Las Fuentes Norte, Aragón Healthcare Service (SALUD), Zaragoza, Spain; ^6^Research Group on Health Services in Aragon (GRISSA), Institute for Health Research Aragón (IIS Aragón), Zaragoza, Spain

**Keywords:** technostress, academic stress, social support, ICT, general health

## Abstract

**Introduction:**

This research aims to study the role of social support as a mediator in the relationship between technostress or academic stress and health in university students.

**Methods:**

A descriptive, quantitative cross-sectional study has been carried out through a self-reported survey answered by 389 students during March and April 2022. The current level of health was the outcome variable. Technostress and academic stress were the criterion variables. Perceived social support was the mediator variable. The sociodemographic variables and ICT use at the educational level were the independent variables.

**Results:**

Women have higher levels of technostress and academic stress than men. Social support significantly and positively mediates the relationship between academic stress and self-perceived health in men.

**Discussion:**

There is a clear need to develop new social management strategies that assist students in developing stable and long-lasting social networks, which can reduce stress during the student period and provide personal tools for later working life.

## Introduction

The World Health Organization (WHO) defines stress as “the set of physiological reactions that prepares the body for action”. In a situation of stress, an automatic reaction is triggered that produces changes at a physiological, emotional, and behavioral level. The sympathetic nervous system is activated, increasing oxygen consumption and heart rate, improving concentration and attention, and inhibiting the parasympathetic system, which suppresses the reproductive and immune systems (Sandín, 2008). Stress has an adaptive function, but its chronicity and intensity have negative repercussions for health, including cognitive, emotional, and physiological symptoms, concentration and memory difficulties, mental fatigue, cognitive overload, anxiety, irritability, burnout, muscle pain, headaches, and insomnia (Tams et al., 2014; Martín Rodriguez, 2020; Galvin et al., 2022; Kasemy et al., 2022).

### Stress and health

University students may experience academic stress, defined as “that which is suffered by students in secondary or higher education and whose exclusive source is stressors related to the activities to be carried out in the school environment” (Barraza and Silerio, 2007). The main academic stressors are exams, presenting assignments, tutorials, work overload, lack of time, competition among classmates, group work, lack of incentives, conflicts with classmates, high demand academic tasks, little autonomy in academic work, and work pressure (Barraza and Silerio, 2007; Gutiérrez Rodas et al., 2010; González Cabanach et al., 2010a). The greatest academic stress is caused by exams, distribution of time, meeting stipulated deadlines, and academic overload (Feldman et al., 2008; González Moreno, 2017; García-García, 2019). Epidemiologically, previous studies estimate that between 47% and 55% of university students suffer from moderate levels of academic stress (Rivas et al., 2014; Estrada et al., 2021).

The consequences of academic stress on health are similar to those of psychosocial stress. The student body can suffer consequences in their physical and psychological health, since the students’ lifestyle is modified by the academic demands and the stress that, in most cases, determines the acquisition of health risk behaviors (i.e., excessive consumption of caffeine, tobacco, stimulants or tranquilizers, hypercaloric, hypersodic or hyperlipidic intake). These lifestyle changes make them susceptible to headaches, sleep disturbances, irritability, lack of concentration, anxiety, depression, and burnout (Extremera Pacheco et al., 2007; Balanza et al., 2009).

### Stress and technostress

Likewise, the university population may be a group vulnerable to the stress generated by the use of information and communication technologies (ICTs) in both academic and personal spheres. In the current university context, the use of ICTs has been consolidated and increased, given that the vast majority of students use laptops, tablets or smartphones in the classroom (González Elices, 2021). The term technostress was used in 1984 by the psychologist Craig Brod to denominate the physical and emotional alterations that some people suffered when faced with handling computers (Jiménez, 2010). It is defined as a negative psychological state that is related to the use of ICTs, exposure to them, or an anticipatory fear or threat of their use in the future (Salanova, 2003). It derives from a mismatch between demands and available resources to use ICTs, which produces a high level of unpleasant psychophysiological activation, tension, and discomfort when using technologies or thinking about their use (techno-anxiety), eventually reaching the point of rejecting ICTs (techno-phobia) (Salanova et al., 2006).

Technostress affects both those with little technological knowledge who may reject the use of ICTs and perceive them as something negative, as well as those accustomed to its use, who experience frustration due to continuous training, recycling and the rapid acquisition of new knowledge (Minaya Lozano, 2008). In addition, it entails consequences for physical and mental health, such as eyestrain, headaches, backaches, digestive problems, irritability, frustration, demotivation, anxiety, memory and concentration problems, addiction, burnout, and lower satisfaction (Tams et al., 2014; Samaha and Hawi, 2016; Mahapatra and Pati, 2018; Rodríguez-Vásquez et al., 2021; Sánchez-Macías et al., 2021; Wang et al., 2021).

It must be taken into account that ICTs alone are “neutral” since they do not themselves generate negative or positive consequences. Just as a person can experience techno-stress when using ICTs, one could also experience flow and engagement; that is, a psychological state in which the user focuses with dedication on that activity with which they experience pleasant sensations and forgets their other thoughts (Sharafi et al., 2006; Barrera-Algarín et al., 2022).

Following the model of interaction between demands and resources proposed by Karasek and Theorell (1990), high levels of technostress are connected to high ICT-related demands and an individual’s lack of technological resources (Salanova, 2003). Along the same lines, the model of interaction between demands, control, and social support would be applicable (Johnson and Hall, 1988). According to this model, social support acts as a modulator of stress if the individual has the quantity and quality of social support they need. If, on the other hand, their social support is scarce or they find themselves in a situation of discrimination or intimidation, social support can become a factor that generates stress.

### Social support, stress, and health

Social support, defined as the degree to which people’s needs for belonging, affiliation, affection, identity, security and approval are satisfied through interaction with others, can directly or indirectly affect stress and health (Clemente, 2003). Social support can promote adaptive health behaviors, provide well-being, and inhibit the negative effects of stress, and its stress-buffering effect is widely accepted by the scientific community (Barra Almagiá, 2004; González Cabanach et al., 2010b).

Thus, 3 models are proposed to explain the association between social support, stress, and health. First, the “direct effect” model, in which social support directly and positively influences health and well-being to the extent that it contributes to satisfying human needs like safety, social contact, belonging, esteem, affection, etc., regardless of the individual’s stress levels (Cohen and Wills, 1985; Pérez Bilbao and Martín Daza, 1997). In the “indirect effect” model, social support can directly reduce work stress levels and improve coping and, therefore, indirectly improve health (Forbes and Roger, 1999; Schwarzer et al., 2004). Finally, in the “buffer effect” model, social support does not have a direct effect on stress or health, but rather allows the stressful situation and coping strategies to be redefined, protecting individuals from the consequent negative effects (Strom and Egede, 2012; Fachado et al., 2013).

In general, previous studies confirm the benefits of social support on health in the university environment. The results indicate how social support is related to a better quality of life, a lower level of academic stress, and better mental health (de Almeida et al., 2018; Roming and Howard, 2019), and present variations according to both gender and source of social support. On the one hand, women tend to prefer emotional support (shows of empathy, love, and trust) over informative support (receiving useful information to deal with the problem) (Forbes and Roger, 1999). On the other, social support from peers and friends is related to better management of mental health and academic stress (Feldman et al., 2008; Muirhead and Locker, 2008; Fernández-González et al., 2015). However, more recent studies find only moderate associations between social support and degree of mental health (Pasinringi et al., 2022), and even negative associations with psychological well-being in women (Luna et al., 2020).

Since the beginning of the COVID-19 pandemic, there has been an increase in the use of ICTs. During that period the classes were held online, through google meet, email and discussion forums. Likewise, the interaction between teachers and students, and among the students themselves, was carried out through video conferences, social networks, and email. This situation could have modified ways of relating and communicating generally, key elements for the generation of social support and its impact on health and stress. That is why it is necessary to look in depth at the process by which social support exerts its effect, in the interest of the human, labor, and social development of university youth.

For this reason, this research aims to study the role of social support as a mediator in the relationship between technostress or academic stress and health in Spanish university students following the COVID-19 pandemic.

## Materials and methods

### Design

A descriptive, quantitative, cross-sectional study was conducted on university students using a self-reported survey.

### Participants and sample size

The study has been carried out with a sample of the university student population over 18 years of age.

According to the Government of Spain and the Ministry of Universities (2021), the total number of students enrolled in the Spanish University System (SUE) in the 2020–2021 academic year was 1,679,518, including Bachelor, Master, and Doctorate students. For a margin of error of 5% and a 95% probability of success, with a confidence level of 95% and an accuracy of 3%, a sample of at least 213 individuals was needed. Once the term for administering the survey was over (April 30, 2022), a final sample of 389 participants was obtained.

The inclusion criteria were the following: individuals over the age of 18, of both sexes, who understood written and spoken Spanish, who have provided their informed consent and who were enrolled in the 2021/2022 academic year in a Bachelor’s, Master’s, or Doctorate program at a Spanish University.

### Instruments

The following instruments were used to collect the information necessary for this study:

We collected sociodemographic variables (sex, age, place of residence, marital status, cohabitation status, type of studies, university enrolled in, and work) through an *ad hoc* questionnaire.

**Technostress questionnaire** (technoanxiety and technofatigue) (created by the Work and OrganizationalNeTwork, WoNT) (Salanova et al., 2006). It consists of 26 items, which evaluate technostress as psychosocial harm with three dimensions: (1) Affective (anxiety vs. fatigue), (2) Attitudinal (skeptical attitude towards technology), and (3) Cognitive (beliefs of ineffectiveness in the use of technology). The items are responded to through a Likert-type frequency scale ranging from “0” (not at all/never) to “6” (always/every day). Obtaining high scores in these three dimensions is an indicator of technostress in its two manifestations: technoanxiety and technofatigue. To diagnose technoanxiety, high scores in anxiety, skepticism and ineffectiveness should be obtained and for technofatigue, high scores in fatigue, skepticism and ineffectiveness. High scores in any of the individual dimension does not necessarily indicate technostress, but it could develop or appear in the future if appropriate measures are not taken. The questionnaire has an adequate internal consistency, exceeding in all cases the minimum Cronbach’s Alpha score of 0.70, which ensures the validity and reliability of the measures (Salanova et al., 2006).

**Goldberg General Health Questionnaire (GHQ-28).** It is subdivided into 4 subscales, with 7 questions each, referring to somatic symptoms, distress/anxiety, social dysfunction, and depression (Lobo et al., 1986). According to Godoy-Izquierdo et al. (2002), the questionnaire has been taken as a positive indicator of current level of health or well-being: the higher the scores, the better the general physical and psychological health. Thus, obtaining a high score in the subscale of “physical state” or somatic symptoms indicates a good level of physical health, in the subscales of “anxiety” and “depression” indicates the absence of anxious and depressive symptoms, and in the subscale of social dysfunction or “daily well-being” indicates that one has the personal capacity to develop a healthy and functional daily life. The responses are presented in Likert format with 4 possibilities (from 0 to 3). The individual scores for each item, which, when added together, form the scores for each subscale, as well as the total (general health) obtained from the sum of the latter, were entered into the statistical analysis. The questionnaire presents good psychometric qualities with a Cronbach’s alpha score of 0.97 for the full scale, 0.93 for somatic symptoms, 0.92 for distress/anxiety, 0.91 for social dysfunction and 0.97 for depression (Godoy-Izquierdo et al., 2002).

**University academic stress questionnaire (CEAU)** (García-Ros et al., 2012). The potential stressors relating to the university environment were studied using a Likert-type scale with 5 possibilities (0 being no stress at all and 5 being very stressful). This questionnaire consists of 21 items grouped into 4 stress-generating factors during the university period: academic obligations, academic record and future prospects, interpersonal difficulties, and expression and communication of ideas. The items in the first factor category have to do with academic obligations that evaluate the level of academic stress in relation to the completion of compulsory tasks and assignments, academic overload, study-related activities, and performance on evaluation tests. The second factor evaluates the stress caused by anticipating future academic situations or problems such as concluding studies within the stipulated time frame, obtaining good grades, maintaining or obtaining a scholarship, or choosing subjects during the degree course. Thirdly, the factor category on interpersonal difficulties evaluates the stress generated by conflicts with professors and classmates, as well as competitiveness with the latter. The last stress-generating factor evaluates the stress produced by presentations of work, participation in class activities such as debates, and dealing with professors during tutoring hours. Thus, a high score indicates the presence of academic stress. The questionnaire presents an adequate internal consistency for the four dimensions, with a Cronbach’s Alpha value of 0.70 (García-Ros et al., 2012).

**Questionnaire to measure the frequency and scope of TIC use at the educational level (CUTIC-28).** This questionnaire evaluates the usefulness of ICTs and the emotion generated by their use or non-use among young university students (Jiménez Rodríguez et al., 2017). The questionnaire collects data on digital behaviors and opinions on the usefulness of ICTs in the educational setting in two media: computer (computer or laptop) or tablet, and cell phone (cell phone). It consists of 28 items distributed into two groups of 14 items and three dimensions. The items in both groups are identical, but one of the groups corresponds to computer or tablet use and the other to cell phone use. The first-dimension groups the items that measure the frequency of ICT use for games, messaging, and social networks; the second measures the usefulness of ICTs in the educational setting in relation to group work, research, classroom work, and information search; the third dimension measures the behavior/emotion generated by ICT: irritability, relaxation, and addiction. Responses are recorded in time frequency intervals (hours per day) or with a 5-point Likert response (from never to always). Following the recommendation of Jiménez Rodríguez et al. (2017), values above 2 in frequency and behavior exceed the mean value; in effect, the average is above 3.5. The questionnaire presents a good internal consistency with an alpha coefficient of 0.86 (Jiménez Rodríguez et al., 2017).

**Questionnaire to measure perceived social support (Spanish version of the Social Support Questionnaire-Short Form)** (Martínez-López et al., 2014). This questionnaire is composed of 6 items, representing moments of stress or need in different situations. For each item, it assesses the number of people that each individual perceives as willing to help and support him or her in a given situation, and the degree of satisfaction with this support. The items relating to the degree of satisfaction are answered on a 6-point Likert scale (very dissatisfied to very satisfied) and the number of people on a 9-point scale, with 1 being one person and 9 being nine or more people. This questionnaire measures two different aspects of perceived social support: availability and the index of satisfaction with perceived availability, calculated by averaging the scores obtained, with a maximum of 36 for the satisfaction score and 54 for availability. The questionnaire presents good psychometric properties with Cronbach’s Alpha figures of 0.89 and 0.94 (Martínez-López et al., 2014).

### Variables

#### Dependent variables

The current level of health (GHQ-28) (Outcome variable). In this case, global health was considered as an independent variable using the complete scale, where the higher the scores, the better the perception of health status.

Technostress and academic stress. Technostress was obtained through the Technostress Questionnaire taking into account the two dimensions (technofatigue and technoanxiety) together, in such a way that the higher the scores, the greater the perception of technostress. Academic stress was obtained by generating a new variable through the sum of the four dimensions collected in the CEAU (academic obligations, academic record and future prospects, interpersonal difficulties, and expression and communication of ideas).

#### Mediator variables

Perceived Social Support was a synthetic variable generated through the Spanish version of the Social Support Questionnaire-Short Form. Given that this index was obtained using the NHS information-gathering technique, we calculated Cronbach’s alpha to verify its reliability in the present sample, obtaining a value of 0.968.

#### Covariables

The independent variables included in the study were: (i) sociodemographic variables: age (it was recorded as a continuous quantitative variable), sex (binary sex, it was recoded as a dummy variable (man/woman)), living together (0-no, 1-yes), studies (0-Postgraduate studies (masters, doctorates and postgraduate studies), 1-Graduate), job (0-not working, 1 working); and (ii) ICT use at the educational level (CUTIC-28) as considered through three dimensions considered as continuous variables: frequency, usefulness, behavior/emotion.

### Procedure

Data collection was carried out employing an online survey, conducted through the Google Forms platform and disseminated through social networks (LinkedIn^1^ and WhatsApp^2^) and institutional distribution lists (the University of Zaragoza and student organizations). Finally, only those surveys that were fully answered were taken into account.

### Ethics

To request participation in the study, the link to the anonymous survey was sent, complying with the regulations in force regarding data protection (Organic Law 3/2018, of December 5, on Personal Data Protection and guarantee of digital rights). Google’s privacy conditions were observed, providing the link to the information and requesting prior approval as a requirement for participation in the study. Participants had to give their informed consent, being free to opt out of the survey at any time. The dissemination period took place during the months of March and April 2022.

This project has been approved by the Research Ethics Committee of the Autonomous Community of Aragon (CEICA) (no. PI22-114) and by the Data Protection Office of the University of Zaragoza (no. RAT 2022–49).

### Data analysis

First, a descriptive analysis (frequencies for categorical variables; means and standard deviation for continuous variables) was carried out to examine the composition of the sample, and the normality or non-normality of the data was tested using the Kolmogorov–Smirnov test with the Lillierfors modification since the sample was greater than 30 cases. Second, in order to see if there were differences between men and women in the study variables, mean differences were studied using Student’s t-test for quantitative variables and chi2 (χ^2^) for qualitative variables. Thirdly, in order to analyze the association between the principal variables of the study (criterion, outcome, and mediator variables), correlations between them were also calculated. Finally, to determine the mediator effect between health and stress variables (technostress and academic stress), various regression analyses were carried out using the procedure designed by Baron and Kenny (1986). This procedure requires that the predictor (stress variables), criterion (health), and mediator variables (perceived social support) be positively correlated with each other. Due to the positive associations observed between health and the covariables (age, living together, studies, job, university), we decided to control for the effects of these variables by entering them in the first step of the regression. To determine whether perceived social support mediates the relationship between self-rated health and stress variables, four mediation analyses were carried out using the Process macro for SPSS (Hayes, 2018), one for each stress variable and others for women and men with the goal of seeing how the variables of interest behave in men and women separately in order to be able to later analyze gender inequalities. The bootstrapping technique with 10,000 subsamples was used to estimate the confidence interval (95%).

Tests were considered significant when *p* < 0.05. The analyses were carried out using IBM SPSS Statistics 25 (IBM Corp, 2017) and Stata 16 (StataCorp, 2019), both licenses from the University of Zaragoza.

## Results

The description of the main study variables is shown in Table 1. A total of 389 subjects participated in the study, of whom 29.8% were men and 70.2% were women. The ages of the sample ranged from 18 to 60 years, with the mean age being 24.93 years. About 85% of the participants in the study resided in Aragón. 73.6% of the men and 67.8% of the women reported being single, although in both sexes around 95% reported living together. Both in technostress and in the two factors that comprise this item (technoanxiety and technofatigue), women registered statistically higher scores than men (*p* < 0.004). Along the same lines, in all four dimensions of academic stress (academic obligations, academic record and future outlook, interpersonal difficulties, and expression and communication of own ideas) it was women who presented higher scores than men, with statistically significant differences (*p* < 0.001). Finally, concerning social support, men registered higher availability and satisfaction, but no statistically significant differences were found between the sexes.

**Table 1:** Sample description.

Variables	Men (*n*, %)	Women (*n*, %)	*p*
Age	26.17 (8.63%)	24.42 (7.47%)	0.043
Residence place			
Aragón	98 (84.5%)	239 (87,5%)	0.055
Others	18 (15.5%)	34 (12.5%)	
Marital status			
Married	30 (25.9%)	84 (30.8%)	0.534
Single	85 (73.3%)	185 (67.8%)	
Separated/divorced	1 (0.9%)	4 (1.5%)	
Living together			
Yes	109 (94%)	254 (93%)	0.909
No	7 (6%)	19 (7%)	
Studies			
Graduate	69 (59.5%)	207 (75.8%)	0.008
Master	14 (12.1%)	14 (5.1%)	
PhD	32 (27.6%)	51 (18.7%)	
Others	1 (0.9%)	1 (0.4%)	
Job			
Yes	59 (59.9%)	118 (43.2%)	0.095
No	57 (49.1%)	155 (56.8%)	
**Self-rated health (GHQ)**, *M* (SD)*	59.1 (11.5)	50.8 (13.3)	0.062
**Technostress**	21.46 (8.10)	23.95 (7.15)	0.003
Technoanxiety	10.37 (4.23)	11.67 (3.79)	0.003
Technofatigue	11.09 (3.98)	12.27 (3.47)	0.004
Academic stress			
Academic obligations	2.876 (0.835)	3.498 (0.821)	<0.001
Academic record and future outlook	2.356 (0.844)	2.976 (0.945)	<0.001
Interpersonal difficulties	1.936 (0.839)	2.293 (1.007)	<0.001
Expression and communication of own ideas	2.413 (0.968)	3.127 (0.960)	<0.001
Social support			
Availability of social support	5.44 (2.172)	5.07 (1.953)	0.099
Satisfaction with social support	5.15 (1.196)	5.07 (1.137)	0.548

*M (SD): mean and Standard Deviation. Health (GHQ-28) (Outcome variable). In this case, global health was considered as an independent variable using the complete scale, where the higher the scores, the better the perception of health status. Stress is measured (Criterion variables). Technostress was obtained through the Technostress Questionnaire taking into account the two dimensions (technoanxiety and technofatigue) together, in such a way that the higher the scores, the greater the perception of techno-stress. Academic stress was obtained by generating a new variable through the sum of the four dimensions collected in the CEAU (academic obligations, academic record and future prospects, interpersonal difficulties, and expression and communication of ideas). Perceived Social Support was a synthetic variable generated through the Duke-UNC questionnaire. Sociodemographic variables: Age: age was recorded as a continuous quantitative variable; Sex (binary sex): sex was recoded as a dummy variable (man/woman); living together (0-no, 1-yes); studies [0-Postgraduate studies (masters, doctorates and postgraduate studies), 1-Graduate]; job (0-not working, 1 working); and TIC use at the educational level (CUTIC-28) as considered through four dimensions considered as continuous variables: frequency, usefulness, behavior/emotion.

In men (Table 2), health correlated negatively and significantly with technostress (*r*: −0.266, *p* < 0.001) and its two constructs technoanxiety (*r*: −0.243, *p* < 0.001) and technofatigue (*r*: −0.282, *p* < 0.001), with Academic stress, including academic obligations (*r*: −0.494, *p* < 0.001), record and future prospects (*r*:−0.360, *p* < 0.001), interpersonal difficulties (*r*: −0.251, *p* < 0.001) and expression and communication of one’s ideas (*r*: −0.370, *p* < 0.001), and with use of TICs including conduct (*r*: −0.262, *p* < 0.001). Finally, health correlated positively and significantly with both constructs of social support (availability of social support (*r*: 0.319, *p* < 0.001) and satisfaction with social support (*r*: 0.382, *p* < 0.001).

**Table 2:** Correlations of the study variables in men.

	1	2	3	4	5	6	7	8	9	9.1	9.2	10.1	10.2	10.3	10.4	11.1	11.2	11.3	12.1	12.2
1. Health	1																			
2.Age	0.099	1																		
3.Residence	−0.048	0.413**	1																	
4.Marital Status	−0.086	−0.275**	−0.039	1																
5. Studies	0.182	0.682**	0.287**	−0.269**	1															
6. Living together	−0.156	−0.360**	−0.185*	0.316**	−0.338**	1														
7. University	−0.034	0.001	0.253**	0.065	−0.068	0.060	1													
8. Job	0.163	0.592**	0.176	−0.342**	0.753**	−0.365**	0.030	1												
9. Technostress	−0.266**	0.010	0.016	0.060	−0.097	0.088	0.173	−0.080	1											
9.1 Technoanxiety	−0.243**	−0.008	0.013	0.076	−0.111	0.095	0.171	0.092	0.986**	1										
9.2 Technofatigue	−0.282**	0.029	0.019	0.042	−0.079	0.078	0.169	−0.063	0.984**	939**	1									
10. Academic stress																				
10.1 Academic obligations	−0.494**	−0.124	0.069	0.069	−0.258**	0.117	0.149	−0.136	0.332**	0.312**	0.343**	1								
10.2 Academic record and future prospects	−0.360**	−0.150	0.129	0.162	−0.106	0.166	0.154	−0.166	0.270**	0.241**	0.285**	0.614**	1							
10.3 Interpersonal difficulties	−0.251**	0.047	0.005	0.095	0.135	0.047	0.076	0.082	0.300**	0.296**	0.294**	0.431**	0.469**	1						
10.4 Expression and communication of one’s own ideas	−0.370**	−0.072	0.199*	0.092	−0.088	−0.009	0.148	−0.085	0.193*	0.183*	0.198*	0.481**	0.416**	0.188*	1					
11. Use of ICTs																				
11.1 Frequency	−0.024	−0.024	0.096	−0.057	0.072	0.094	−0.040	0.018	−0.002	0.008	−0.012	0.097	0.049	−0.064	0.042	1				
11.2 Utility	0.060	−0.336**	0.027	−0.030	−0.288**	−0.089	−0.060	−0.197*	−0.165	−0.166	−0.158	0.165	0.070	−0.120	−0.030	−0.025	1			
11.3 Conduct	−0.262**	−0.071	0.160	0.013	−0.053	0.036	0.206*	−0.026	0.040	0.024	0.056	0.170	0.184*	0.122	0.146	0.109	0.132	1		
12. Social support																				
12.1 Availability of social support	0.319**	−0.109	−0.134	0.068	−0.082	0.058	0.020	−0.055	−0.061	−0.071	−0.048	−0.202*	−0.2071*	−0.186*	−0.354**	−0.067	0.188*	−0.120	1	
12.2 Satisfaction with social support	0.382**	0.051	−0.006	−0.044	0.121	−0.044	−0.115	0.012	−0.067	−0.064	−0.069	−0.201*	−0.246**	−0.077	−0.280**	0.046	0.219*	0.163	0.500**	1

Current level of health (GHQ-28) (Outcome variable). In this case, global health was considered as an independent variable using the complete scale, where the higher the scores, the better the perception of health status. Stress is measured (Criterion variables). Technostress was obtained through the Technostress Questionnaire taking into account the two dimensions (technoanxiety and technofatigue) together, in such a way that the higher the scores, the greater the perception of technostress. Academic stress was obtained by generating a new variable through the sum of the four dimensions collected in the CEAU (academic obligations, academic record and future prospects, interpersonal difficulties, and expression and communication of ideas). Perceived Social Support was a synthetic variable generated through the Duke-UNC questionnaire. Sociodemographic variables: Age: age was recorded as a continuous quantitative variable; Sex (binary sex): sex was recode as dummy variable (man/woman); living together (0-no, 1-yes); studies [0-Postgraduate studies (masters, doctorates and postgraduate studies), 1-Graduate]; job (0-not working, 1 working); and TIC use at the educational level (CUTIC-28) as considered through four dimensions considered as continuous variables: frequency, usefulness, behavior/emotion.

Similarly, for women (Table 3), health correlated negatively and significantly with technostress (*r*: −0.264, *p* < 0.001) and its two constructs technoanxiety (*r*: −0.256, *p* < 0.001) and technofatigue (*r*: −0.265, *p* < 0.001) with Academic stress, including academic obligations (*r*: −0.399, *p* < 0.001), record and future prospects (*r*: −0.358, *p* < 0.001), Interpersonal difficulties (*r*: −0.265, *p* < 0.001) and expression and communication of one’s ideas (*r*: −0.239, *p* < 0.001), and with the use of ICTs including conduct (*r*: −0.283, *p* < 0.001). Finally, health correlated positively and significantly with both constructs of social support [availability of social support (*r*: 0.166, *p* < 0.001) and satisfaction with social support (*r*: 0.184, *p* < 0.001)].

**Table 3:** Correlations of the study variables in women.

	1	2	3	4	5	6	7	8	9	9.1	9.2	10.1	10.2	10.3	10.4	11.1	11.2	11.3	12.1	12.2
1. Health	1																			
2.Age	0.064	1																		
3.Residence	0.067	−0.002	1																	
4.Marital Status	0.028	−0.209**	−0.006	1																
5. Studies	0.052	0.485**	0.041	−0.123*	1															
6. Living together	−0.107	−0.427**	−0.043	−0.096	−0.311**	1														
7. University	0.062	−0.020	0.340**	0.050	−0.016	−0.036	1													
8. Job	0.014	0.565**	0.018	−0.062	0.668**	−0.405**	0.119*	1												
9. Technostress	−0.264**	−0.022	−0.064	0.113	−0.109	0.039	−0.085	−0.065	1											
9.1 Technoanxiety	−0.256**	−0.019	−0.065	0.106	−0.112	0.043	−0.097	−0.063	0.986**	1										
9.2 Technofatigue	−0.265**	−0.025	−0.060	0.117	−0.101	0.033	−0.069	−0.064	0.983**	0.940**	1									
10. Academic stress																				
10.1 Academic obligations	−0.399**	−0.112	0.034	0.024	−0.176**	0.057	−0.089	−0.068	0.250**	0.231**	0.262**	1								
10.2 Academic record and future prospects	−0.358**	−0.291**	0.004	0.046	−0.113	0.148*	−0.056	−0.160**	−0.172**	0.163**	0.176**	0.548**	1							
10.3 Interpersonal difficulties	−0.265**	−0.163**	−0.004	−0.052	−0.022	0.020	0.027	−0.044	0.136*	0.134*	0.134*	0.441**	0.425**	1						
10.4 Expression and communication of one’s own ideas	−0.239**	−0.215**	−0.004	−0.001	−0.109	0.166**	−0.112	−0.183**	0.231**	0.223**	0.233**	0.370**	0.276**	0.191**	1					
11. Use of ICTs																				
11.1 Frequency	−0.095	0.026	0.037	0.030	0.014	−0.079	−0.024	−0.024	0.015	0.026	0.003	0.034	0.221*	0.114	0.039	1				
11.2 Utility	0.030	−0.039	0.098	−0.055	−0.104	0.023	0.009	−0.027	−0.118	−0.109	−0.124*	−0.163**	0.079	0.084	0.051	0.064	1			
11.3 Conduct	−0.283**	−0.198**	−0.038	0.042	−0.115	0.187**	−0.031	−0.191**	0.110	0.127*	0.087	0.160**	0.291**	0.191**	0.211**	0.232**	0.194**	1		
12. Social support																				
12.1 Availability of social support	0.166**	0.008	0.016	0.006	0.044	0.009	0.059	0.003	−0.032	−0.034	−0.029	−0.126*	−0.184**	−0.158**	−0.189**	−0.156**	0.051	−0.087	1	
12.2 Satisfaction with social support	0.184**	0.072	0.016	−0.083	0.088	−0.033	0.043	0.083	−0.076	−0.074	−0.077	−0.090	−0.136*	−0.149*	−0.086	−0.155*	0.140*	−0.232**	0.420**	1

Current level of health (GHQ-28) (Outcome variable). In this case, global health was considered as an independent variable using the complete scale, where the higher the scores, the better the perception of health status. Stress is measured (Criterion variables). Technostress was obtained through the Technostress Questionnaire taking into account the two dimensions (technoanxiety and technofatigue) together, in such a way that the higher the scores, the greater the perception of technostress. Academic stress was obtained by generating a new variable through the sum of the four dimensions collected in the CEAU (academic obligations, academic record and future prospects, interpersonal difficulties, and expression and communication of ideas). Perceived Social Support was a synthetic variable generated through the Duke-UNC questionnaire. Sociodemographic variables: Age: age was recorded as a continuous quantitative variable; Sex (binary sex): sex was recode as dummy variable (man/woman); living together (0-no, 1-yes); estudies [0-Postgraduate studies (masters, doctorates and postgraduate studies), 1-Graduate]; job (0-not working, 1 working); and TIC use at the educational level (CUTIC-28) as considered through four dimensions considered as continuous variables: frequency, usefulness, behavior/emotion.

### Mediation analysis

**Figure 1:**
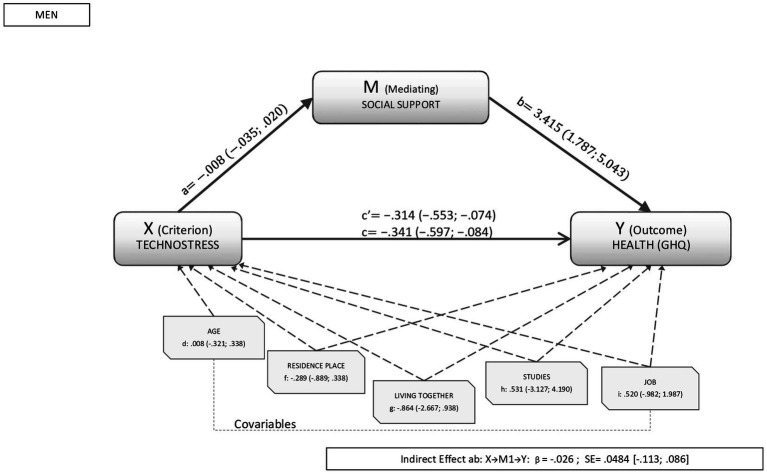
Results of the mediation analysis in men. Outcome variable: self-rated health; criterion variable: technostress; and mediating variable: social support, where a, b, c, d, f, g, h, and i are the direct effects of the mediation (c’ is the standardized direct effect). Indirect effects are represented by β_social support_.

**Figure 2:**
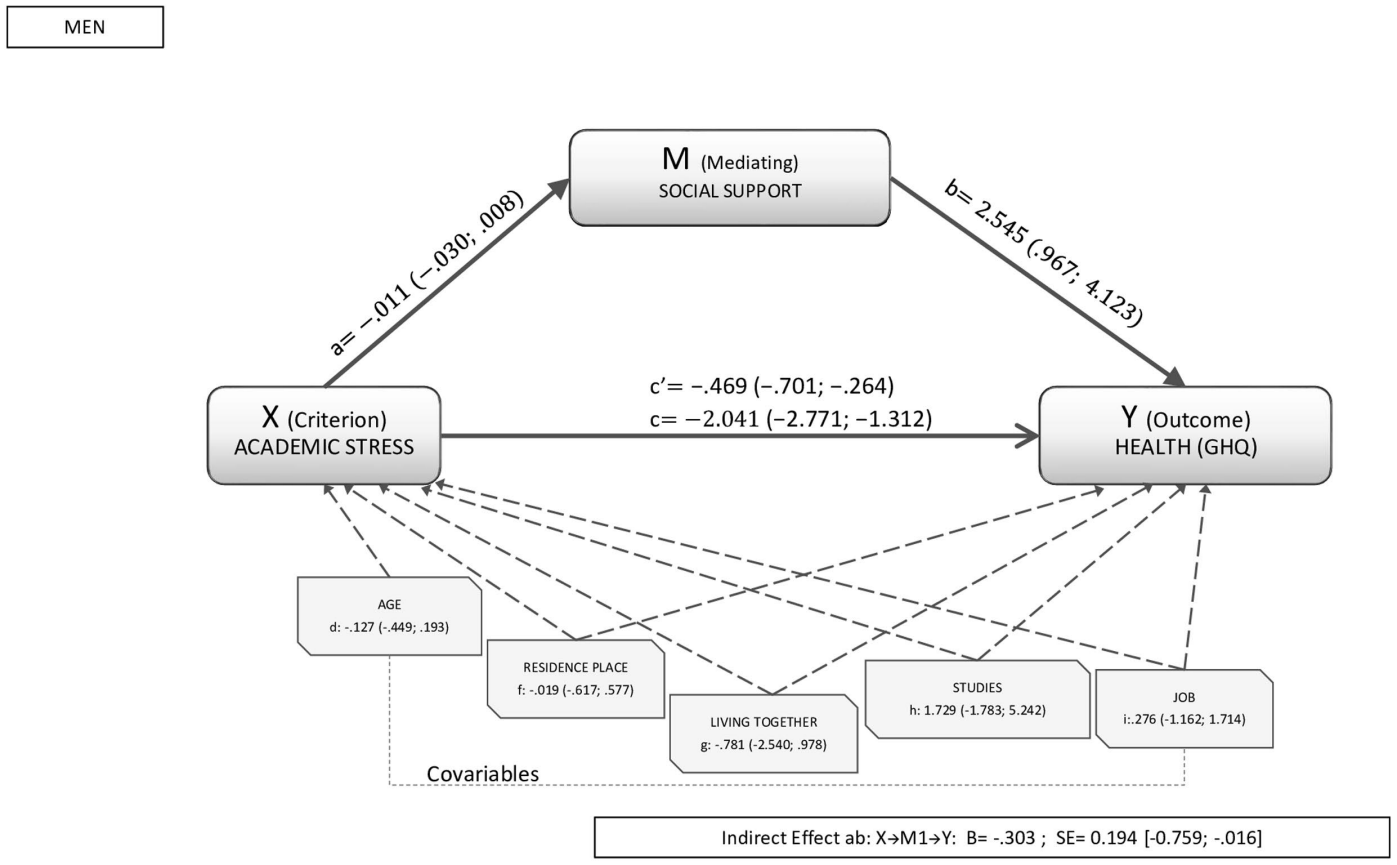
Results of the mediation analysis in women. Outcome variable: self-rated health; criterion variable: technostress; and mediating variable: social support, where a, b, c, d, f, g, h, and i are the direct effects of the mediation (c’ is the standardized direct effect). Indirect effects are represented by β_social support_.

The percentage of variance explained by social support in the relationship between technostress and health oscillated between 10% to 20% in both sexes when the stressor analyzed was technostress. As shown in Figures 1, 2 for men and women respectively, the analyses revealed a direct significant effect between technostress and health (β_men_: −0.341, CI95%: −0.553; −0.074; β_women_: −0.483, CI95%: −0.701; −0.266). Similarly, the direct effects of the mediator variable (social support) on health were significant in both sexes (β_men:_ 3.415, CI95%: 1.787; 5.043 β_women_:1.915, CI95%: 0.571; 3.260).

In the analysis of the global model, the indirect effect of technostress on health was not significant in either sex (β_men_: −0.026, CI95%: −0.113; 0.086; β_women_: −0.020, CI95%: −0.084; 0.016). The covariables analyzed were also not found to influence the relationship between technostress and health in either sex.

**Figure 3:**
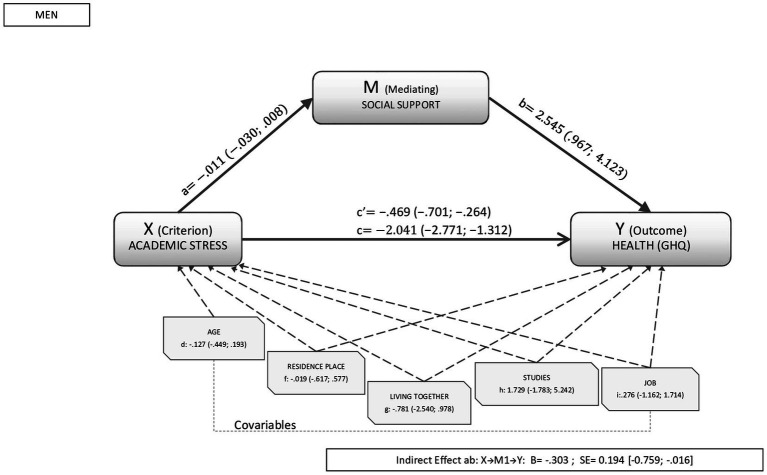
Results of the mediation analysis in men. Outcome variable: self-rated health; criterion variable: academic stress; and mediating variable: social support, where a, b, c, d, f, g, h, and i are the direct effects of the mediation (c’ is the standardized direct effect). Indirect effects are represented by β_social support_.

**Figure 4:**
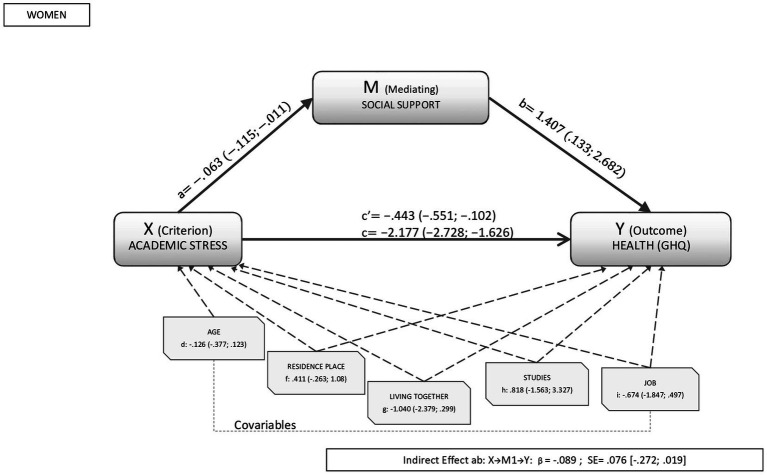
Results of the mediation analysis in women. Outcome variable: self-rated health; criterion variable: academic stress; and mediating variable: social support, where a, b, c, d, f, g, h, and i are the direct effects of the mediation (c’ is the standardized direct effect). Indirect effects are represented by β_social support_.

When we took academic stress into account, the percentage of variance explained by social support in the relationship between academic stress and health oscillated between 10% to 30% in both sexes. As shown in Figures 3, 4 for men and women respectively, the analyses revealed a direct significant effect between academic stress and health (β_men_: −0.469, CI95%: −0.701; −0.264; β_women_: −0.443, CI95%: −0.551; −0.102). Similarly, the direct effects of the mediator variable (social support) on health were significant in both sexes and (β_men:_ 2.545, CI95%: 0.967; 4.123 β_women_:1.407, CI95%:0.133;2.682).

In the analysis of the global model, the indirect effect of academic stress on health was significant in men (β_men_: −0.303, CI95%: −0.759; −0.016); but not in women (β_women_: −0.089, CI95%: −0.272; 0.019). The covariables analyzed were also not found to influence the relationship between academic stress and health in either sex.

## Discussion

This research aimed to study the mediator effect of social support on the relationship between technostress or academic stress and health in university students after the COVID-19 pandemic in Spain.

From the data analyzed, we observed that participation in this study was higher among women than among men. In regard to self-perceived health, although we did not find statistically significant differences between men and women, we did observe a certain tendency for men to have higher scores than women, i.e., they had a better perception of their health than women. In both technostress and academic stress, women recorded higher scores than men. In the availability of social support and satisfaction with social support, men reflected higher scores than women although no statistically significant differences were observed between the sexes. Finally, mediation analyses showed that in both men and women, social support did not influence the relationship between technostress and health. However, the mediation analyses did show how social support exerted a positive influence on the relationship between academic stress and health, a fact which was not observed in women.

In the present study, greater participation was observed by women than by men, in line with what happened in studies from other universities (Benavente-Cuesta and Quevedo Aguado, 2018; de la Rosa Gómez et al., 2018). The age of the participants was around 25 years, relatively high since undergraduate studies generally begin at 18 years of age and end at 22/23 years of age. The increase in the average age of the participants in this study may be related to the fact that 21% of the participants in the sample were doctoral students. Likewise, an increase in the starting age of university studies has been observed; in Spain, in the 2021/2022 academic year, 25% of new students were between 22 and 25 years of age (Ministerio de Universidades, 2021). The present data is consistent with the data provided by the Organisation for Economic Co-operation and Development (OECD) report entitled “Education Overview 2019” where it is shown that in Spain there is a higher proportion of women who reach Higher Education in the OECD countries, 15% more than that of men. Likewise, the OECD report also reveals the increase in education level in people over 25 years of age, which is also consistent with the present results (González Merino, 2020).

The results showed the presence of some differences in health perception between men and women (Aguilar-Palacio et al., 2018; Sánchez-Recio et al., 2021). Throughout the scientific literature, we can find several studies that show how women in different contexts, the academic (student) environment and the workplace, for example, report having worse self-perceived health than men (Marco-Ahulló et al., 2021; Porru et al., 2022). In Spain there has been an important change in the educational level of the population in recent decades, mainly among women, who have gone from having a medium or low level of education to a high level (Herrera Ceballos, 2014; Busemeyer and Garritzmann, 2017). This is positively related to women’s perception of health, which, although worse than that of men, has improved compared to previous periods (Urbanos-Garrido and González López-Valcárcel, 2015; Aguilar-Palacio et al., 2018). On the other hand, many gender factors influence women to continue to report poorer health, such as the glass ceiling (Arcaya et al., 2015; García-Calvente et al., 2015). Many women, when they start university studies, think that they will be able to achieve the same professional development as men and later realize that it is still difficult for them to do so (Taparia and Lenka, 2022). On the other hand, in Spain, even today, despite the new trends of co-responsibility and newly defined masculinities, it is still women who work double and triple time between carrying full-time paid work and the main burden of family care, which continues to have a direct impact on their health (Casado, 2021; García-de-Diego and García-Faroldi, 2022). At the university level, numerous studies show that it is women who suffer more stress (in all its dimensions), have a poorer capacity for adaptation and resilience, and have higher levels of self-demand, a fact that may also justify the presence of gender differences in the perception of health status in this age group (DeSalvo et al., 2006; Morales-Rodríguez, 2021). Finally, in that vein, numerous studies show that it was university women who suffered a greater impact on their health during this period, as well as greater stress and greater coping difficulties (Wang et al., 2020; Hoyt et al., 2021; Marco-Ahulló et al., 2021).

The results reflect how women register higher scores in technostress and academic stress than men. Several previous studies show this. On the one hand, women are more likely than men to suffer from technostress and academic stress (García-Ros et al., 2012; Lemos et al., 2018; Barrera-Herrera et al., 2019). On the other hand, in general they perceive higher levels of social support from family and friends (Day and Livingstone, 2003; Talwar et al., 2013). In the present study, no statistically significant differences were found between the sexes, who both report adequate levels of social support.

Although there are currently few studies that analyze the moderating capacity of social support, its stress-buffering effect is widely accepted by the scientific community (González Cabanach et al., 2010a). It is through the buffer effect that social support changes the relationship between stress and health, redefining the stressful situation, favoring coping and protecting subjects from the negative effects of stress (Strom and Egede, 2012). Previous research has shown how family social support moderates stress and depression (Suwinyattichaiporn and Johnson, 2022) and how peer support is a protective factor in dealing with stressful situations (Vega Valero et al., 2017). However, at present, it is unknown to what degree each mechanism contributes to the global effect that social support exerts on health (Strom and Egede, 2012).

The results of this study show how social support does not mediate the relationship between technostress and health in either sex. This could be related to what was described by Moran Paucar (2019), who recently observed that students perceived greater social support on the network (Internet) than from friends. On the other hand, this same author showed how less social support (from family and friends) was negatively correlated with stress (mainly psychological stress) (Moran Paucar, 2019). Likewise, other current studies show how low levels of stress and family social support were significantly related to moderate and high levels of quality of life (Roming and Howard, 2019), even as the relationship between social support and the degree of mental health is at moderate levels (Pasinringi et al., 2022).

It has been observed how new ways of relating through ICTs and social networks are being produced, which could be related to the fact that social support does not moderate the relationship between technostress and self-perceived health. These new ways of relating make social ties weaker and more immediate, easy to create but with low commitment and little satisfaction (Moreno-Colom, 2009; Durán Heras and Rogero García, 2009). Similarly, the COVID-19 pandemic has had a direct impact on ways of relating in society in general and particularly in the student population. As a result of the confinement, the need arose to create new teaching spaces through ICTs, which meant an increase in autonomous work by the student through virtual tools.

Concerning academic stress, the results show that social support significantly and positively mediates the relationship between academic stress and self-perceived health in men, findings that were not observed in women. These results may be in line with those previously described by other authors, in which it was observed that female students present higher levels of stress than men, as well as a greater probability of being affected by the stress of the people around them (García-Ros et al., 2012; Matud, 2017; Lemos et al., 2018). This may mean that they need higher levels of social support to cushion the stressor and control the negative impact on their health since women have a smaller coping and resilience capacity than men (de la Rosa Gómez et al., 2018; Vizoso Gómez, 2019; Morales-Rodríguez, 2022). However, despite high levels of academic stress, women have better academic performance and greater well-being in the educational field than men (González-Cantero et al., 2020). Also, it could be possible that there are other psychological variables with a greater capacity to moderate stress than social support. Recent previous studies on university students have detected how social support, satisfaction and resilience present differentiated levels, evolution and relationships based on gender and academic year (Zhang et al., 2018; Hu et al., 2020).

### Limitations

The present study is not without limitations. As we used non-probabilistic snowball sampling, it would be interesting to use a random sampling method in order to generalize the outcomes. Since the questionnaire was disseminated online, the representativeness of the sample could not be controlled and people without internet access are automatically excluded (Arroyo Menéndez and Finkel, 2019). Due to the cross-sectional nature of the study is difficult to establish causal relationships and the associations identified might be difficult to interpret (Wang and Cheng, 2020).

It would be advisable to carry out a study with a sample that includes other universities with a greater offer of online degrees, since the University of Zaragoza, excepting the period of confinement due to the pandemic, is a face-to-face university. In addition, to extend the sample to various countries since most of the respondents belong to one of the four university campuses of the University of Zaragoza, distributed throughout the Autonomous Community of Aragón. Likewise, the starting age of the studies completed should be analyzed, since the average age is relatively high (25 years), as should the course that the participants are taking. It also must be taken into account that, in the present study, no differentiation has been made between in-person social support and social support through ICTs.

The present study is one of the first to analyze how social support mediates the relationship between technostress and academic stress and self-perceived health, which is why the data must be interpreted with caution and more multi-center studies with larger samples are necessary to be able to corroborate these results. In addition, there are other variables such as personality traits and resilience, which could also influence the present results (Conti et al., 2018; Zhang et al., 2018; Hu et al., 2020).

## Conclusion

While the process by which social support has an effect remains unclear, it is extremely important to deepen the investigation of these aspects, in pursuit of the human, labor, and social development of university youth. Therefore, it would be necessary to carry out studies differentiated by gender that measure the impact on the self-perceived health of men and women at the same level of technostress and academic stress; as well as the inclusion of variables such as resilience.

There is a clear need to develop new social management strategies that help students to create stable and lasting social networks, which can moderate stress during the student period, as well as personal tools to face later working life.

## Data availability statement

The raw data supporting the conclusions of this article will be made available by the authors, without undue reservation.

## Ethics statement

To request participation in the study, the link to the anonymous survey was sent, complying with the regulations in force regarding data protection (Organic Law 3/2018, of December 5, on Personal Data Protection and guarantee of digital rights). Google’s privacy conditions were observed, providing the link to the information and requesting prior approval as a requirement for participation in the study. Participants had to give their informed consent, being free to opt out of the survey at any time. The dissemination period took place during the months of March and April 2022. This project has been approved by the Research Ethics Committee of the Autonomous Community of Aragon (CEICA) (no. PI22-114) and by the Data Protection Office of the University of Zaragoza (no. RAT 2022-49). The studies were conducted in accordance with the local legislation and institutional requirements. The participants provided their written informed consent to participate in this study.

## Author contributions

AA-M, AA-L, and RS-R contributed to the study with the original idea and design, data interpretation and approval of the final version. BM, SG-S, and MS-C contributed to the writing of the article and the acquisition and interpretation of data. All authors contributed to the article and approved the submitted version.

## Funding

This work was supported by the Aragonese Primary Care Research Group (GAIAP, B21_23R) which is part of the Department of Innovation, Research and University at the Government of Aragón (Spain); and the Institute for Health Research Aragón (IIS Aragón).

## Conflict of interest

The authors declare that the research was conducted in the absence of any commercial or financial relationships that could be construed as a potential conflict of interest.

## Publisher’s note

All claims expressed in this article are solely those of the authors and do not necessarily represent those of their affiliated organizations, or those of the publisher, the editors and the reviewers. Any product that may be evaluated in this article, or claim that may be made by its manufacturer, is not guaranteed or endorsed by the publisher.

## Footnotes

^1^ http://www.linkedin.com

^2^ https://whatsapp.com
